# A Co-Designed Active Video Game for Physical Activity Promotion in People With Chronic Obstructive Pulmonary Disease: Pilot Trial

**DOI:** 10.2196/23069

**Published:** 2021-01-27

**Authors:** Joshua Simmich, Allison Mandrusiak, Stuart Trevor Smith, Nicole Hartley, Trevor Glen Russell

**Affiliations:** 1 Faculty of Health and Behavioural Sciences The University of Queensland Brisbane Australia; 2 School of Health and Human Sciences Southern Cross University Coffs Harbour Australia; 3 Faculty of Business, Economics and Law The University of Queensland Brisbane Australia

**Keywords:** fitness trackers, chronic obstructive pulmonary disease, physical activity, video games, smartphone, mobile phone

## Abstract

**Background:**

People with chronic obstructive pulmonary disease (COPD) who are less active have lower quality of life, greater risk of exacerbations, and greater mortality than those who are more active. The effectiveness of physical activity interventions may facilitate the addition of game elements to improve engagement. The use of a co-design approach with people with COPD and clinicians as co-designers may also improve the effectiveness of the intervention.

**Objective:**

The primary aim of this study is to evaluate the feasibility of a co-designed mobile game by examining the usage of the game, subjective measures of game engagement, and adherence to wearing activity trackers. The secondary aim of this study is to estimate the effect of the game on daily steps and daily moderate-to-vigorous physical activity (MVPA).

**Methods:**

Participants with COPD who were taking part in the co-design of the active video game (n=9) acted as the experiment group, spending 3 weeks testing the game they helped to develop. Daily steps and MVPA were compared with a control group (n=9) of participants who did not co-design or test the game.

**Results:**

Most participants (8/9, 89%) engaged with the game after downloading it. Participants used the game to record physical activity on 58.6% (82/141) of the days the game was available. The highest scores on the Intrinsic Motivation Inventory were seen for the value and usefulness subscale, with a mean of 6.38 (SD 0.6). Adherence to wearing Fitbit was high, with participants in both groups recording steps on >80% of days. Usage of the game was positively correlated with changes in daily steps but not with MVPA.

**Conclusions:**

The co-designed mobile app shows promise as an intervention and should be evaluated in a larger-scale trial in this population.

## Introduction

### Background

Chronic obstructive pulmonary disease (COPD) is a leading cause of death worldwide [1] and is associated with persistent respiratory symptoms, reduced exercise capacity, and poorer quality of life [2,3]. People with COPD are generally less active than people without COPD [4,5], and physical activity levels generally decline as the disease progresses [6].

Inactivity in people with COPD is associated with poorer health-related outcomes, including a lower quality of life, greater risk of exacerbations, and greater mortality [6-10]. International guidelines promote regular physical activity in people with COPD, generally targeting 30 min of moderate physical activity on most days [11].

Interventions that are effective in achieving this targeted level of physical activity in people with COPD are limited [12,13]. Pulmonary rehabilitation, involving supervised exercise training, is strongly recommended for people with COPD [14,15], as it is very effective at increasing exercise capacity [16,17]. Despite this, many people with COPD struggle to maintain their physical activity levels in the months after pulmonary rehabilitation [18], and some do not become more active at all [19]. A longer period of pulmonary rehabilitation may be more effective at improving activity levels, with one systematic review finding that all studies showing no impact of exercise training on physical activity had durations of less than 12 weeks, whereas all interventions lasting longer than 12 weeks improved physical activity levels [12]. However, lengthening the duration of pulmonary rehabilitation beyond the 12 weeks may lead to reduced availability of places in pulmonary rehabilitation programs, which are already limited in many countries [20].

Consumer-grade electronic pedometers, such as those developed by Fitbit (Fitbit Inc), have been shown to be valid devices for measuring physical activity in people with COPD [21] and may enable people with COPD to be more conscious of their physical activity levels. Behavioral interventions that use technology such as wearable pedometers to facilitate self-monitoring of physical activity have shown some short-term effectiveness in improving physical activity levels in people with COPD [22,23]. However, the benefits of physical activity may be short lived, possibly because of poor long-term engagement with these interventions [24]. A recent Cochrane review of technology-based COPD self-management interventions concluded that “researchers also must take into consideration strategies that will promote long-term engagement with smart technology” [22].

Gamification is an emerging strategy to improve engagement with digital technology, including within the context of health care [25]. Gamification is the use of game design elements in nongame contexts [26] and is a common feature in health and fitness apps, including fitness tracker apps [27,28]. Most commonly, the apps include game features such as digital rewards for goal attainment, avatars (visual representations of players), social or peer pressure (including leaderboards), and the provision of feedback on performance. However, little research exists on game interventions paired with wearable activity trackers in people with COPD, and trials of gamified interventions in other populations have shown conflicting results. For example, a trial in healthy adolescents of an activity tracking website known as *Zamzee* (Zamzee Co) demonstrated a 54% increase in moderate-to-vigorous physical activity (MVPA) over 6 weeks [29], but a trial *Active Team (Portal Australia)*, a gamified smartphone app for healthy adults*,* had no effect on objectively measured MVPA over 3 months [30]. Although both interventions in these studies were gamified, they differed substantially in the game elements that were used [29,31], underscoring the impact that different designs can have on the effectiveness of gamification.

Active video games (AVGs), defined as video games that require physical activity to play [32], are another approach that has been used in an attempt to increase physical activity. A number of studies have investigated the use of AVGs in COPD and other chronic respiratory conditions, showing that they can evoke a similar physiological response to more traditional exercises (eg, stationary bicycle) while being more enjoyable [33]. However, the effect that AVGs have on habitual physical activity in an unsupervised setting has not been extensively studied in respiratory disease populations [34] or older adults [35]. In addition, the studies to date have generally used commercially available AVGs that are designed for the general population rather than for older adults [36] or to address the preferences of people with chronic diseases. Trials of AVGs in older adults with chronic diseases, such as COPD, are required, and such trials might be expected to demonstrate greater adherence or effectiveness if those AVGs are designed to take into account the needs and preferences of the patient population involved in the trial.

### Aims

Using a co-design process with people with COPD and clinicians, we developed an AVG called *Grow Stronger* to promote physical activity in people with COPD after pulmonary rehabilitation. A co-design methodology known as participatory design was used. Participatory design is a research and design practice where the users of a particular system participate as co-designers throughout the design process rather than merely as testers providing feedback to designers [37]. A participatory design process can, at least in some circumstances, improve the effectiveness of serious games for health [38].

The primary aim of this study is to evaluate the feasibility of the *Grow Stronger* AVG intervention in people with COPD by assessing the usage of and engagement with the AVG, along with adherence to wearing the Fitbit activity tracker. The secondary aim of this study is to assess the effect of the Grow Stronger AVG, when combined with a Fitbit activity tracker and Fitbit app, on physical activity in comparison to the Fitbit activity tracker and Fitbit app alone. Primary outcomes included usage of the AVG in the experiment arm of this pilot trial (how often the AVG was used, what types and difficulties of activity goals were chosen, and what breathlessness values were reported), subjective measures of engagement with the AVG in the experiment group, and adherence to wearing Fitbit activity trackers in both the experiment and control arms. Secondary outcomes were daily steps and daily physical activity levels in both the experiment group and control group, as assessed by the Fitbit activity monitor.

## Methods

### Overview

This study is a pilot trial nested within an iterative co-design process to develop an AVG. This co-design process comprised a series of focus groups with people with COPD (n=10) and clinicians (n=18), aiming to outline, design, and develop an AVG. For the trial, 9 of the 10 people with COPD who were taking part in the co-design process comprised the experiment group, who received the AVG app in addition to a Fitbit activity tracker and Fitbit app. The control group comprised individuals with COPD who did not take part in the co-design process, who received only the Fitbit activity tracker and the Fitbit app.

The study was approved by the Prince Charles Hospital Human Research Ethics Committee and ratified by the University of Queensland Human Ethics Research Office.

The co-design process took place between June 2019 and November 2019, with the pilot trial being conducted for 3 weeks at the end of this process, from October 4, 2019 to October 25, 2019.

### Participants

People who reported they had been clinically diagnosed with COPD were recruited. A letter containing information about the study was sent to recent (previous 12 months) attendees of pulmonary rehabilitation programs across 4 sites operated by Queensland Health in the Moreton Bay Region of Queensland, Australia. Interested potential participants were screened to ensure they met the inclusion and exclusion criteria. Participants were included if they had attended pulmonary rehabilitation in the past 12 months, were able to read and speak English, and were able to exercise independently (with or without the use of mobility aids and supplemental oxygen). Participants were excluded if they did not have access to a smartphone, were unable to exercise because of medical or physical limitations, required 24-hour supplemental oxygen, or lacked the visual acuity to view the text displayed on typical mobile devices.

### Procedures

After all participants were recruited, a randomized sequence of participants was generated. Participants were alternately allocated into 2 groups: (1) an active co-design group (hereafter the experiment group), which took part in the focus groups and received an activity monitor and the AVG intervention and (2) a control group, which received an activity monitor but did not take part in focus groups or received access to the AVG. For the 19-week duration of the co-design process, participants in both the experiment and control groups were provided with a consumer-grade wearable activity monitor, namely, a Fitbit Alta HR or Fitbit Charge HR 2 (Fitbit Inc.). This activity monitor was paired to the participant’s smartphone and was capable of tracking steps, physical activity, and heart rate. Participants in both groups were provided with instructions on how to use the Fitbit app, and participants in both groups were set up as friends with other participants within their group, allowing participants to see the weekly step total of other participants and access other social features. It was not possible to blind the participants to their group allocation.

**Figure 1:**
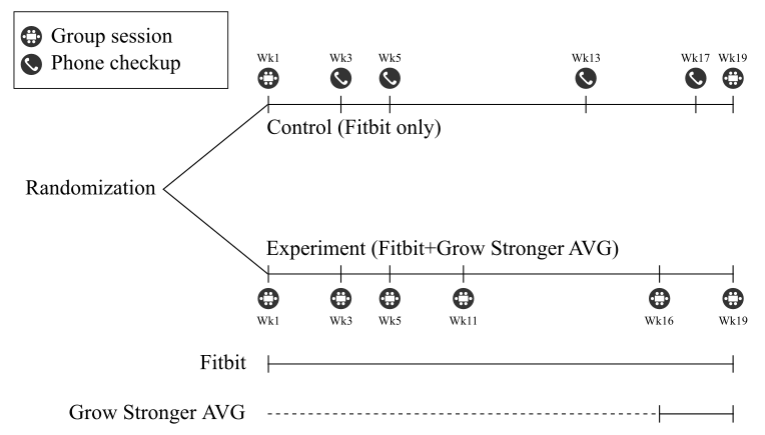
Contact times, types of contact, and interventions received for each group. Group sessions for the experiment group comprised focus groups in the co-design process along with an install session in week 16, whereas the 2 group sessions for the control group were a group enrolment session in the first week and a study conclusion session in the final week. AVG: active video game.

The control group did not participate in the focus groups and only had in-person contact with the research team during a group enrollment session and study conclusion session. As per Figure 1, the control group received regular telephone check-ins across the trial duration to set appropriate step goals on the Fitbit app and to provide the same opportunity to raise any device-related issues as was afforded the experiment group before and after the focus groups. Participants in the experiment group were able to trial the test version of the AVG during the final 3 weeks of the development process. However, not all participants were able to download and use the app the day it became available, resulting in some participants having a shorter period to experience the AVG than others. At the conclusion of the trial, participants relinquished their wearable fitness trackers, but those using the app continued to have access to it for at least a month after the conclusion of the trial.

### Game Intervention

The *Grow Stronger* game and the co-design process undertaken to develop it will be described more fully elsewhere. In brief, *Grow Stronger* is a smartphone app that functions as both a game and a physical activity diary. Progress in the game requires the player to report the completion of upper body and lower body physical activities commonly used in the physical rehabilitation of people with COPD. The game features a simple stick figure image of each activity, and players are provided with an additional handout with more complete instructions for each activity. Each day, players choose an upper body and lower body activity and set at what difficulty or intensity they wish to perform these activities. At the completion of each activity, users must report their perceived Borg breathlessness value using a slider present in the app to receive their reward for that activity.

The game features 2 parallel game modes, which can be used together or separately. The first mode functions as a single player mode and uses the theme of growing a garden, where players are rewarded with water in a watering can that can be used to grow a potted plant. The second game mode functions as a cooperative multiplayer game mode and has the theme of a caravan trip around Australia, visiting multiple well-known Australian destinations. As a team, players are rewarded with progress on the trip, determined by the average number of activities completed by the team. All data from the use of the game are reported to a web interface that allows clinicians to monitor the progress of all players and sends encouraging messages. A more complete description of the game, along with representative screenshots and a full list of all available activities, is available in Multimedia Appendix 1.

### Outcome Measures

Several primary outcome measures were collected by the AVG in the experiment group, namely, the usage of the app, type of activities completed, difficulty level selected by participants for each activity, and reported Borg breathlessness ratings for each physical activity. Adherence to wearing the Fitbit activity tracker was assessed using step data collected from the Fitbit devices, with nonwear defined as zero steps recorded for an entire day.

Additional primary outcome measures of subjective game engagement were collected at the conclusion of the study by asking the experiment group to complete 3 questionnaires. First, the Game Engagement Questionnaire (GEQ) was used [39], which was measured on a three-level scale (1=no, 2=maybe or sort of, 3=yes). Second, 5 subscales of the intrinsic motivation inventory (IMI) were employed: interest or enjoyment, perceived competence, effort or importance, value or usefulness, and relatedness [40]. Each of these subscales was measured on a seven-point Likert scale (1=completely disagree to 7=completely agree). Finally, a cognitive processing and cognitive activation (CPCA) questionnaire was developed for use in this study, adapted from Hollebeek et al [41] and measured on a 7-point Likert scale as for the IMI. All questions were given using either paper-based or web-based forms immediately after the final focus group.

Secondary outcome measures collected from all participants included total steps and duration and the intensity of physical activity. These measures were automatically collected for the entire 19-week duration of the study by the Fitbit activity trackers provided to participants in both the experiment group and the control group. Devices such as these are considered to be valid low-cost devices to measure physical activity in people with COPD [21]. MVPA was assumed to be the sum of the 2 highest Fitbit categories for active minutes (*fairly active* and *very active* categories). This approach has been previously used when comparing consumer-level activity monitors to research-grade accelerometers, demonstrating moderate-to-strong validity for MVPA measured by Fitbit devices in healthy adults in free-living conditions [42].

Before the first focus group, participants also filled in a prestudy survey, providing information on their gender, age, employment status, confidence in technology (on a 0-10 scale), and degree of self-perceived functional limitation because of breathlessness, as assessed using the Medical Research Council (MRC) dyspnea scale [43].

### Data Analysis

Data were analyzed and visualized using Python (Python 3.7; Python Software Foundation). Step counts and minutes of activity were collated to a daily figure for each participant, which was used to compute each participant’s average for steps per day and MVPA per day over the period before and after the AVG was downloaded. Days where no step data were recorded were ignored when calculating each participant’s average steps per day and MVPA per day, effectively interpolating these missing days with the participant’s own average for that period. One participant in the control group did not wear the Fitbit during the final 3 weeks of the study and so was excluded from the pre-post comparisons of steps per day and MVPA per day.

Owing to the small sample size and nonnormality evident in some outcomes, the Spearman rank correlations were used to examine relationships between outcome measures (ie, pre-post change in daily steps, change in MVPA, game engagement on GEQ and IMI scales, and game usage). The Spearman rank correlation is not affected by skewness and generally copes better with light-tailed distributions than the Pearson correlation [44].

As this was a pilot randomized controlled trial, with a sample size determined by optimum focus group size during the co-design process rather than being adequately powered to detect differences in primary outcome measures, no statistical tests were performed, and data are presented as mean and SD only.

## Results

### Participants

**Figure 2:**
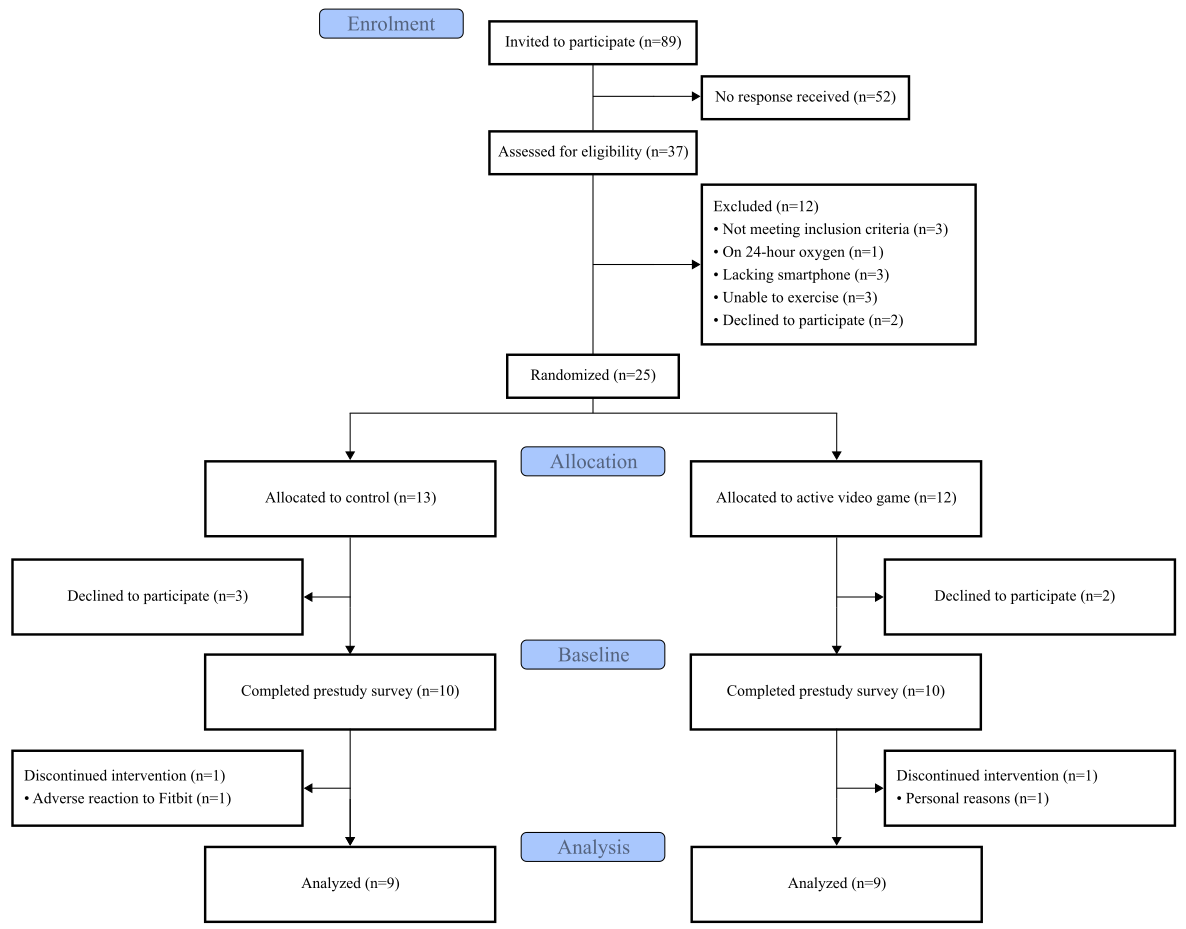
Consolidated Standards of Reporting Trials diagram showing the flow of participants through the study.

Figure 2 shows the progression of participants throughout the study in a Consolidated Standards of Reporting Trials diagram. Of the 89 participants invited to participate in the study, 37 responded and were screened against the inclusion and exclusion criteria. The 25 eligible and consented to participate were randomized into the 2 arms of the study. Of these, 10 from each group attended the first session and completed the prestudy survey. Overall, 2 participants discontinued and withdrew from the study, both during week 4. One participant withdrew for personal reasons, whereas the other withdrew because of reported skin irritation from the Fitbit device. Two other participants reported some skin irritation, resulting in low Fitbit adherence, but did not withdraw from the trial.

One Fitbit device had to be replaced during the trial because of issues with synchronization between the activity tracker and phone, but data were not lost. A number of participants also experienced issues with Bluetooth synchronization, but these issues were resolved after troubleshooting discussions with the research team, and this did not appear to result in a loss of data for any full day for any participant (although loss of part of the data for that day may have occurred).

The results from the prestudy survey for the control and experiment groups are shown in Table 1. Aside from the gender balance, there were no obvious differences between the groups. Both groups had an MRC dyspnea score between grade 2 and grade 3, indicating moderate functional limitation because of breathlessness.

**Table 1:** Details of participants who received the full intervention in each arm of the study (n=9).

Group	Control	Experiment
Female, n (%)	6 (67)	5 (56)
Age (years), mean (SD)	65 (7)	70 (6)
Retired, n (%)	8 (89)	9 (100)
Confidence with technology (0-10 scale), mean (SD)	5.2 (2)	5.3 (2.3)
Medical Research Council dyspnea, mean (SD)	2.4 (1.1)	2.4 (1.2)

### Game Usage Statistics

#### Game Usage Frequency

**Figure 3:**
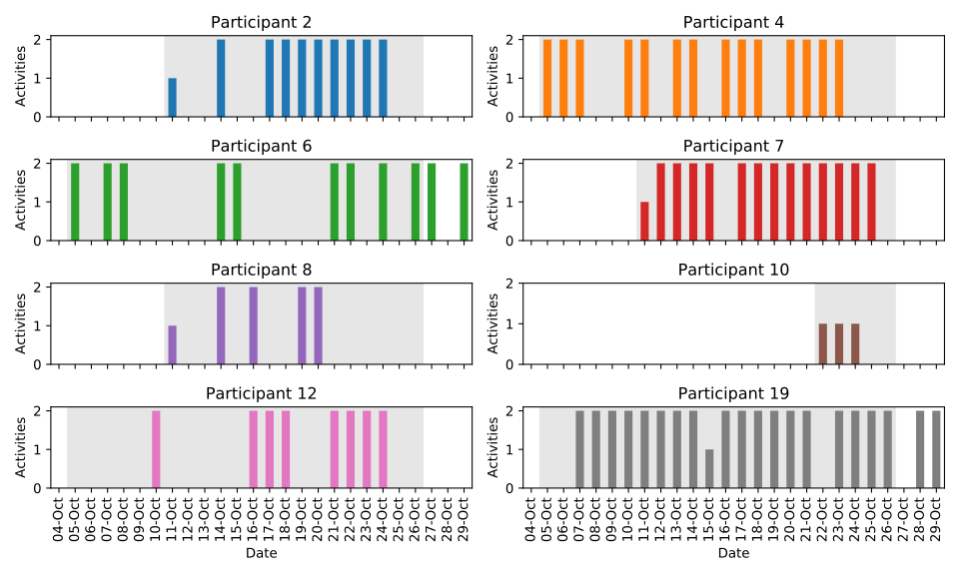
Number of activities recorded per day for each participant in the experiment group. The period where each participant had downloaded the game, but before the test period had concluded, is indicated by the shaded background. One participant was not shown, as they did not use the game after downloading it on their phone.

The number of activities logged per day by each participant in the experiment group is shown in Figure 3. The game allowed a maximum of 2 activities to be recorded each day. Most participants (8/9, 89%) engaged in the game after downloading it. Excluding participants who did not use the game at all, the remaining participants logged at least one activity on 58.6% (82/141; SD 21%) of the days when they had access to the game during the test period. Note that not all participants downloaded and installed the game on their smartphone on the same date. Although the test period concluded on October 25, some participants continued to use the game after this date.

#### Types of Activities Recorded

**Figure 4:**
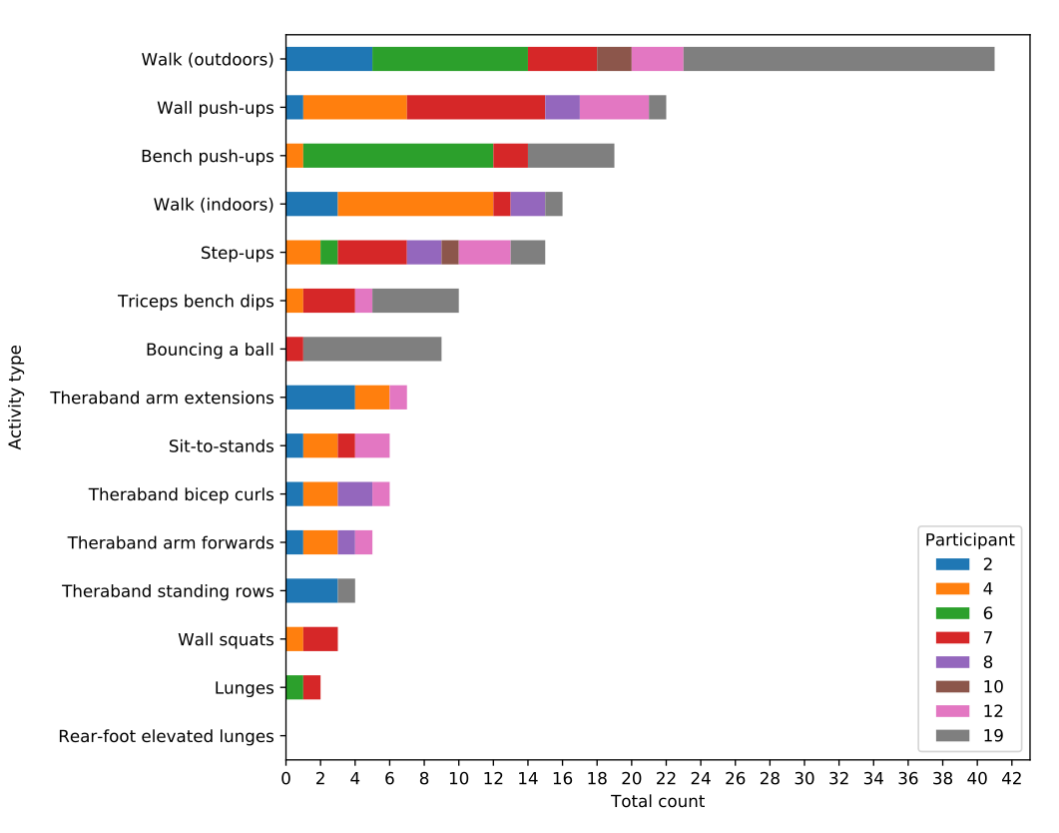
Number of times each activity was recorded by participants in the experiment group.

Figure 4 shows the frequency of activities that were recorded using the app as well as which participants recorded which activity. Outdoor walking was by far the most recorded activity, recorded 41 times. Walking either indoors or outdoors represented 34.5% (57/165) of all recorded activities.

#### Borg Breathlessness Values

The frequencies of Borg breathlessness values, reported on the 0 to 10 modified Borg breathlessness scale, after activities when using the app are shown in Table 2. The mean Borg breathlessness score was 3.8 (SD 1.3).

**Table 2:** Frequency for Borg breathlessness values recorded by the experiment group.

Breathlessness value (modified Borg scale)	Number of events recorded in the app
1	8
2	18
3	32
4	65
5	34
6	3
7	3
8	1
10	1

#### Difficulty Values Selected

The frequency at which participants selected the various difficulty or intensity options in using the app is shown in Table 3. Higher numbers represented greater difficulty for a given physical activity task. All difficulties were roughly equally represented, with no evident skew to higher or lower difficulties.

**Table 3:** Frequency for difficulty values selected by the experiment group.

Selected difficulty	Number of events recorded in the app
1	36
2	47
3	36
4	46

### Game Engagement

People with COPD in the experiment group completed 3 measures of subjective game engagement: IMI, GEQ, and a series of CPCA questions developed for this study.

The scores on the IMI subscales are shown in Table 4. The highest scores were seen for the value and usefulness subscale, with a mean of 6.4 (SD 0.6). All other subscales had lower scores, ranging from approximately 5.1 to 5.7.

**Table 4:** Intrinsic Motivation Inventory subscale scores (7-point Likert scale).

Subscale	Score, mean (SD)
Interest and enjoyment	5.4 (0.5)
Perceived competence	5.7 (1.0)
Effort and importance	5.3 (0.9)
Value and usefulness	6.4 (0.6)
Relatedness	5.1 (1.1)

The total score for the 19 items of the GEQ for each participant is presented in Table 5. The GEQ score totals ranged from 19 to 39, with a mean GEQ total score of 30.4 (SD 6.9). As this scale has a minimum possible score of 19 and a maximum possible score of 57, a score of 30.4 represents 30% (11.4/38) of the distance between these extremes.

**Table 5:** Total scores for the Game Engagement Questionnaire.

Participant	Game Engagement Questionnaire score
2	28
4	36
6	35
7	26
8	30
10	39
12	19

The results for each individual question in the CPCA questionnaire are presented in Table 6. Participants generally had a moderate to high degree of agreement across all questions, ranging from 5.2 to 6.5 on a 7-point Likert scale. Mean scores were higher for items relating to their health goals (items 1-3), all of which had a mean of 6.5 (SD 0.8).

**Table 6:** Cognitive processing and cognitive engagement individual item results.

Question	Score, mean (SD)
CPCA1^a^: Using the game gets me to think about my health goals (n=8)	6.5 (0.8)
CPCA2: I think about my health goals a lot when I'm using the game (n=8)	6.5 (0.8)
CPCA3: Using the game stimulates my interest to learn more about achieving my health goals (n=8)	6.5 (0.8)
CPCA4: I spend a lot of time using the game, compared to other ways of being physically active (n=8)	5.4 (1.4)
CPCA5: Whenever I'm trying to be more active, I usually use the game (n=5)	5.6 (1.5)
CPCA6: The game is what I usually play when I think about being physically active (n=5)	5.2 (2.4)

^a^CPCA: cognitive processing and cognitive activation.

### Fitbit Wear Adherence

**Figure 5:**
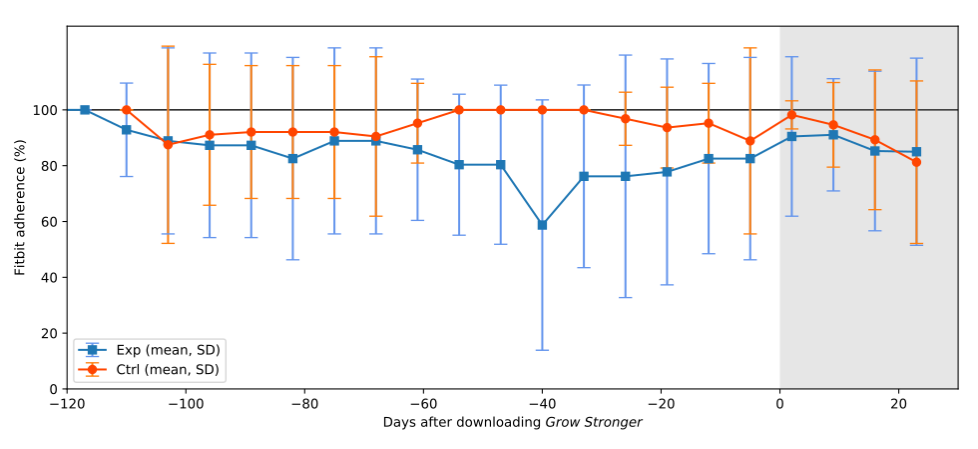
Average weekly adherence to wearing Fitbits in each group before and after the Grow Stronger app was downloaded by the experiment group. The period where each participant had downloaded the game is indicated by the shaded background. The control group, which did not download the game, were aligned with the majority of the experiment group for ease of comparison. As weeks are assumed to start on Mondays, but most participants downloaded the game on a Friday (day 0), the value for the final week before the game was downloaded (the last week in the unshaded area) included values from days 1 and 2 after the game was downloaded. All participants are included in the data presented in this figure. Ctrl: control; Exp: experiment.

Figure 5 shows the weekly average adherence to Fitbit activity trackers for participants across the study period, as calculated by days with a nonzero step count divided by total days and expressed as a percentage. Overall, participants in the control group had a slightly higher average adherence than the experiment group, wearing the Fitbit on 94.5% (1069/1131) of days compared with 84.3% (975/1157) of days in the experiment group. This was especially evident during the middle of the study period when adherence in the experiment group decreased for several weeks.

### Steps

**Figure 6:**
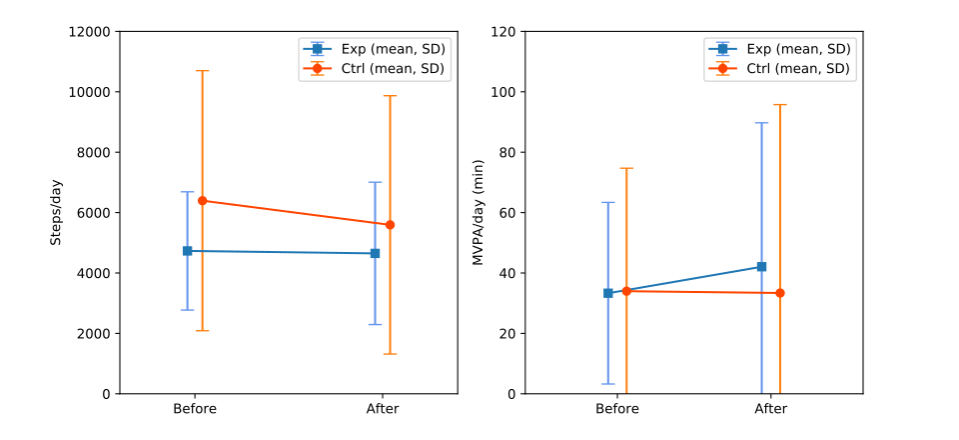
Average steps per day and MVPA per day in each group before and after the game was downloaded. Ctrl: control; Exp: experiment; MVPA: moderate-to-vigorous physical activity.

Across all weeks before the game intervention was downloaded, the experiment group averaged 4730 (SD 1959, range 1493-7522) steps per day, as shown in Figure 6. Steps in the experiment group in the weeks after downloading the *Grow Stronger* AVG averaged 4649 (SD 2357, range 1853-8130) per day, representing a decrease of 81 steps per day or a 2% decrease. In the period before the experiment group downloaded *Grow Stronger,* the control group was averaging 6394 (SD 4306, range 2700-15,000) steps per day, which then decreased by 800 steps per day (800/6394, 12.5%) to 5593 (SD 4277; range 1924-14,367) steps per day.

Individual step count charts for each participant are shown in Multimedia Appendix 2 for the experiment and control groups.

### MVPA

As shown in Figure 6, before the game intervention was downloaded, the experiment group was averaged 33 (SD 30; range 3-76) min of MVPA per day, and in this period, the control group had an average daily MVPA of 34 (SD 41; range 3-120) min. During the game intervention, the experiment group was averaging 42 (SD 48; range 2-122) min of MVPA, and the control group was averaging 33 (SD 62; range 1-182) min of MVPA each day. This represented an increase of approximately 9 min per day or a 26% increase for the experiment group and an approximately 1 min or 2% decrease for the control group per day.

### Correlations Between Outcome Measures

To explore the relationships within and between the secondary outcome measures (pre-post change in MVPA and steps) and the primary outcome measures (game adherence, game engagement on GEQ, and game engagement on IMI), the Spearman correlation coefficients were calculated. Table 7 shows the results in the form of a correlation matrix (a scatter matrix for these comparisons can be found in Multimedia Appendix 3). There appeared to be a moderately high positive correlation, with a Spearman rank correlation coefficient of 0.62 between the pre-post change in daily step and the usage of the *Grow Stronger* app (as assessed by percentage of days during the test period with at least one activity logged). Physical activity was weakly correlated with game usage. The total score on the GEQ correlated moderately strongly and positively with the mean score on the IMI, with correlation coefficients of 0.61. The pre-post change in daily steps appeared to be strongly negatively correlated with both the subjective measures of game engagement, with correlation coefficients of −0.79 for the GEQ and −0.71 for the IMI. The subjective measures of game engagement (GEQ or IMI) did not appear to correlate with changes in daily MVPA or to game adherence.

**Table 7:** Spearman rank correlation matrix between primary and secondary outcomes in the experiment group.

Outcome measures	Spearman rank correlation				
	Δ^a^Moderate-to-vigorous physical activity per day	ΔSteps per day	Game usage	Game Engagement Questionnaire	Intrinsic Motivation Inventory
ΔModerate-to-vigorous physical activity per day (pre-post)	1.00	—^b^	—	—	—
ΔSteps per day (pre-post)	0.21	1.00	—	—	—
Game usage (percentage of days with activities logged on Grow Stronger )	0.45	0.62	1.00	—	—
Game Engagement Questionnaire	0.21	−0.79	0.07	1.00	—
Intrinsic Motivation Inventory	0.19	−0.71	−0.19	0.61	1.00

^a^Δ: change in.

^b^—: These cells are deliberately left blank. The table is symmetrical about the diagonal, so the data are not repeated.

## Discussion

### Summary of Primary Findings

This study assessed the feasibility of the AVG intervention by measuring usage of the AVG in the experiment arm of this pilot trial and adherence to wearing Fitbit activity trackers in both the experiment and control arms. The usage of the game in this study was moderate, with participants who used the game recording at least one activity on 58.6% (82/141) of the days. This is slightly lower than the usage rates reported in the first few weeks of similar studies of apps or websites for physical activity promotion. For example, 68% of users of the gamified *Active Team* app were accessing the gamified app each day by day 7, although this fell to 31% by day 91 [45]. However, usage of *Grow Stronger* was measured by days with logged activities; therefore, it may be expected to be lower than a usage measurement based on days where the intervention was merely accessed. The difference between these 2 methods of measuring usage was illustrated by the pilot trial of *ActivityCoach* (Roessingh Research and Development), which found that participants visited the website an average of 23 times over 4 weeks but filled in the daily symptom diary just 16 times on average [46]. Similarly, in the pilot trial of *Condition Coach* (Roessingh Research and Development), which included a website with a symptom diary and exercise module, the website was visited on 86% of the days available, but the exercise module was only completed on 21% of the days [47]. Although the pilot trial lasted 9 months compared with just 3 weeks in this study, adherence to the *Condition Coach* exercise portal was clearly low throughout: only 5 of the 12 participants completed any exercises in the exercise portal. In comparison, 8 of the 9 participants completed at least one activity in *Grow Stronger*. However, these other apps were able to send notifications to participants during these trials, whereas our *Grow Stronger* app lacked the ability to provide notifications, which may have improved usage if it was available.

The Fitbit was worn on 84% of the days by participants in the experiment group and on 94% of the days by participants in the control group. These values are comparable with those reported by Wan et al [48], where pedometer wear adherence ranged between 80% and 90% in people with COPD. It is worth noting, however, that this pedometer was worn on the hip, pocket, or with a lanyard, in contrast to the wrist-worn Fitbits provided in this study. Adherence is thought to be higher for wrist-worn activity trackers than hip-worn in adolescents [49], although several participants in our study reported skin irritation, which may have decreased wear adherence.

Difficulty options selected in the app by participants were roughly equally distributed, indicating that the difficulty options presented were adequate for participants with COPD. Reported Borg breathlessness values were clustered around 3 to 5 on the modified Borg scale, indicating moderate shortness of breath and representing an appropriate target for exercise in people with COPD [50].

Engagement with the game in the experiment group as assessed on the GEQ averaged 30.4, out of a minimum possible score of 19 and a maximum possible score of 57. This is comparable with the GEQ results originally reported during the development of the GEQ, which were generally around 30 to 32 for adolescents and undergraduates playing common video games [39]. However, the result in this study is lower than the score given by healthy adults for AVGs [51], which would have corresponded to a score of 42 if the adapted GEQ with a seven-point scale used in that study was transformed into the three-point scale used in this study and in the original development study of the GEQ. The scores on the GEQ found in this study may be lower than that seen by other games because several items on the GEQ assess the subjective experiences of immersion and flow that may not be elicited by a game, such as *Grow Stronger*, which does not provide real-time feedback to players. GEQ results for games similar to *Grow Stronger* are unfortunately not available for comparison.

Participants in the experiment group appeared to have given a higher average rating for the value and usefulness subscale of the IMI than other subscales such as interest or enjoyment, relatedness, and effort or importance. This would suggest that participants were primarily motivated to play *Grow Stronger* because they saw value in it rather than because they enjoyed the game, felt a degree of relatedness to other players, or were motivated to put effort into it. It is possible that by being involved in the design of the game from its inception, participants in this trial were especially aware of the value or potential usefulness of the game. In addition, it has been shown that multiplayer AVGs offer greater relatedness than single player ones [52], so designing *Grow Stronger* to provide the option of a single player experience in addition to multiplayer may have diluted ratings on the relatedness dimension.

The CPCA scale showed somewhat higher mean scores for health goal questions than physical activity questions, indicating that participants primarily associated the game with achieving health goals rather than performing physical activity. This could be because of the design of the game, which allowed players to perform physical activity away from their smartphone and then to return to their phone to play *Grow Stronger*. This temporal asynchrony between being physically active and playing the game may have decreased the association between the game and physical activity, whereas participation in the design process may have increased the association between playing the game and reflecting on health goals. As this scale was significantly modified for the purposes of this trial, no comparisons to other studies are available.

It is also noteworthy that a response rate of around 41% (37/89) was observed among participants invited to participate in this study. Although we cannot determine the reason for nonresponse, some may not have responded because of lack of access to, or reservations about, technological interventions. A recent survey of people with COPD attending pulmonary rehabilitation in metropolitan areas of Australia found that 48% had personal access to a smartphone and 57% felt that their technology skills were adequate or better [53]. Nonetheless, the results of that survey and the engagement of participants in our study suggest that a substantial portion of people with COPD are willing to use technology in their rehabilitation.

### Summary of Secondary Findings

In addition to the primary findings mentioned previously, this study also examined the effect on daily steps and daily physical activity levels of the combination of the *Grow Stronger* AVG and Fitbit activity tracker with the Fitbit app, compared with the Fitbit activity tracker with the Fitbit app alone, in patients with COPD. A noteworthy finding of this study was that the experiment group performed an average of 9 min more MVPA after downloading the game, whereas the MVPA in the control group remained roughly similar. Although these results must be interpreted cautiously because of the low sample size of this pilot trial and the large sample SD in the results, this nonetheless suggests that the AVG may have a positive effect on physical activity in this population. To the best of our knowledge, no published research has examined the effect on physical activity of a mobile game intervention combined with a wearable activity tracker versus an activity tracker alone in patients with COPD.

Another secondary finding of this study was that the average steps per day decreased by 13% in the control group but only by 2% in the experiment group. Despite the large variability in this small sample, this finding is consistent with the hypothesis that AVG could ameliorate the decline in physical activity often experienced after pulmonary rehabilitation [18]. It is also possible that the activity tracker itself caused an initial increase in daily steps, which slowly waned over the duration of the study before being partly counteracted by the effect of the AVG in the experiment group. Other studies of people with COPD have, however, examined the combination of activity trackers with mobile apps to encourage daily steps [23]. These mobile apps were not considered games and lacked clear game features but did incorporate behavior change techniques (such as self-monitoring, goal setting, and social support) that could have an effect similar to game design elements. For example, Moy et al [54] and Wan et al [48] compared the effect of a pedometer alone to the same pedometer combined with a website intervention, which encouraged incremental goal setting, allowed social communication between users, and provided educational and motivational messages. These studies, respectively, showed steps per day increased 13% in the intervention group, with no significant change in the control group at 17 weeks [54], and an increase of 19% in steps per day in the intervention group versus a decrease of 5% in steps per day in the control group after 13 weeks [48]. These 2 studies demonstrated an increase in the steps for the intervention with little change to the control group, whereas we found little change in the experiment group but a decrease in the control group. A decrease in daily steps in the control group was also observed in a trial of a mobile app that enabled clinician feedback combined with activity tracker compared with activity tracker alone at 3 or 6 months [55]. However, in contrast to this study where daily steps decreased only in the control group, in that study, both the control group and the experiment group decreased steps per day by approximately 14%. Vorrink et al [55] suggested that either the use of a smartphone app rather than a website or the involvement of health professionals could have contributed to the lack of difference between groups, but the *Grow Stronger* intervention used in this pilot trial was also a mobile app and also involved clinicians, yet a difference between groups was observed. Furthermore, although both Moy et al [54] and Vorrink et al [55] used activity tracking websites or apps that were specifically designed for users with chronic diseases, only the intervention used in a study by Vorrink et al [55] consulted people with COPD during the design phase and only after the initial design had been developed [56].

The observed change in daily steps was positively correlated with game usage, indicating that those who used the game more often also had a greater increase (or smaller decrease) in daily steps. This is consistent with walking, which is the most commonly recorded activity in the game. A correlation between game usage and MVPA was also present but weaker than that between steps and game usage. A stronger correlation between game usage and physical activity may have been expected, given that a recent study found that those in the top quartile for the usage of a gamified smartphone app, *Active Team,* increased their daily physical activity by 18 min (around 17% of baseline), whereas users in the lowest 3 quartiles of app usage decreased their daily physical activity by 8 min (around 8% of baseline) [45]. Physical activity in that study was assessed by research-grade accelerometry rather than the wearable activity tracker provided to participants, as was used in this study. It is possible that Fitbit activity trackers did not count toward the MVPA measurement of the short bouts of strength training that the *Grow Stronger* app encouraged participants to do.

Surprisingly, subjective game engagement, as measured on the GEQ and IMI, appeared to be negatively correlated to steps but not correlated to MVPA. This is contrary to the implicit hypothesis that the engaging nature of AVGs would encourage both more steps and more physical activity. This also conflicts with the results of other AVGs in other populations such as children, where an increased level of intrinsic motivation and enjoyment were correlated with increased physical activity [57]. It is possible that those who found *Grow Stronger* most enjoyable were more likely to have performed other forms of physical activity encouraged by the app, such as upper limb strength exercises, in place of their usual walking. If strength training could not be readily detected as MVPA by the Fitbit, this would not appear as a correlation between game engagement and MVPA. Possible reasons for the negative relationship between subjective game engagement and physical activity remains speculative, as no other studies have examined this relationship in the context of either smartphone AVGs or AVGs for older adults.

Subjective measures of game engagement did not appear to be correlated with game usage, which is contrary to a previous study that demonstrated a correlation between the IMI rating of a smartphone game to improve physical activity and the usage of said game [58]. However, the correlation in that study was assessed before and after a 24-week intervention in a group of 18 people with diabetes. At just 3 weeks with only 9 people with COPD, this study may not have been long enough for a correlation between game usage and subjective game enjoyment to arise or be large enough to detect whether such a correlation did exist. In addition, that study [58] employed a linear regression rather than the Spearman correlation as used in this study, precluding direct comparison between the 2 studies.

### Limitations

This study is limited in several ways. As a pilot trial with a small number of participants and short duration, with the sample size, and trial duration oriented around the co-design process, this trial was underpowered to detect differences in steps and physical activity, which could be expected from such a short intervention. A larger and longer duration trial would be required in the future to gain a meaningful estimate of the effect of the AVG on daily steps and physical activity. In addition, participants in the experiment group were co-designers of the intervention, so they may have been more invested in the game that they helped to create. Therefore, this study’s results may not be generalizable to a population who are naïve to the game.

Furthermore, the trial period formed part of the design process, with feedback from participants used to develop a final version of the game intervention beyond the test version trialed in this study. Therefore, the results of this study do not account for the effect that these revisions may have had on the effectiveness of or adherence to the final version of the game, which will be tested in future studies. For instance, the version of the *Grow Stronger* app used in this study was not able to record usage data regarding when participants accessed the app for purposes other than to record the completion of an activity. As such, no data were available on how often participants used the pause button feature or used the app to interact with one another or check their individual or team progress. Similarly, as mentioned, the app was unable to send notifications, and app usage was likely lower than it would be with reminder notifications enabled. The future version of the app will address these limitations.

As this pilot trial was embedded within a co-design process, the control condition was selected without full knowledge of the eventual design of *Grow Stronger* and so may not have been an ideal comparison. The control group received Fitbit activity trackers and the Fitbit app under the assumption that the AVG provided to the experiment group would most closely resemble the Fitbit app, albeit in the form of a game. However, as a result of the co-design process, *Grow Stronger* more closely resembled a mobile exercise diary. As such, future studies aiming to explore the effect of the game elements of *Grow Stronger* on physical activity should compare this game with a mobile exercise diary app that functions similar to *Grow Stronger* but lacks game elements. In addition, the use of any digital technologies in COPD may not represent the usual standard of care for people with COPD. Therefore, future research may also compare *Grow Stronger* with usual care of people with COPD after pulmonary rehabilitation, namely, providing a control group with an entirely unsupervised home exercise program.

In this study, activity outcome measures were recorded by commercial-grade Fitbit activity monitors, rather than a research-grade physical activity monitor, which presents 2 main limitations. First, although older hip-worn Fitbit devices have been shown to have a good correlation with research-grade activity monitors [21] in people with COPD, this study was conducted with newer wrist-worn Fitbit activity monitors for which such validity data are not known in COPD. The wrist-worn Fitbit Charge 2 devices used in this study have shown high validity for step counts but only moderate validity for MVPA when compared with research-grade accelerometry in older adults [59]. Second, the Fitbit devices were part of the intervention given to both groups, and data from such devices were visible to participants, which may have caused participants to alter their physical activity in response. Future studies may therefore benefit from employing accelerometers with established validity for MVPA and concealing such measurements from participants.

It is also worth noting that the CPCA questions used herein, although they were based on previously validated scales, were modified significantly to fit the purposes of this study and so may no longer retain their validity. Future research should seek to validate these measures or use alternative validated measures.

Finally, no objective tests of lung function or functional status were performed, nor were results from such tests available for this trial. Although it is very likely that all participants had COPD, as they all had attended a pulmonary rehabilitation class that requires referral by a physician, future research may benefit from confirmation of a clinical COPD diagnosis. In addition, the results of spirometry or exercise tolerance tests could be used to appropriately stratify participants in a future trial.

### Conclusions

To our knowledge, this is the first study that trials an AVG designed by people with COPD and clinicians to maintain or enhance physical activity levels. Although the results are limited because of the small sample size, this study is an initial demonstration of the potential value of an app that facilitates physical activities for people with COPD. Future work is required to further improve adherence and to investigate the long-term effects of this intervention. Despite this, the *Grow Stronger* app shows promise as an intervention worthy of a larger-scale trial in this population.

## Appendix

Multimedia Appendix 1 Screenshots and description of the Grow Stronger smartphone app.

Multimedia Appendix 2 Daily step counts for individual participants in the experiment group and control group.

Multimedia Appendix 3 A scatter matrix for correlations between the primary and secondary outcome measures in the experiment group.

## Abbreviations

AVG: active video game
COPD: chronic obstructive pulmonary disease
CPCA: cognitive processing and cognitive activation
GEQ: Game Engagement Questionnaire
IMI: Intrinsic Motivation Inventory
MRC: Medical Research Council
MVPA: moderate-to-vigorous physical activity
