# Supplementary material for: A Co-Designed Active Video Game for Physical Activity Promotion in People With Chronic Obstructive Pulmonary Disease: Pilot Trial
Source: JMIR Serious Games. 2021 Jan 27;9(1):e23069. doi: 10.2196/23069 (PMC7875701; doi:10.2196/23069)
Supplement: Multimedia Appendix 1 [file games_v9i1e23069_app1.docx]

# Multimedia Appendix 1


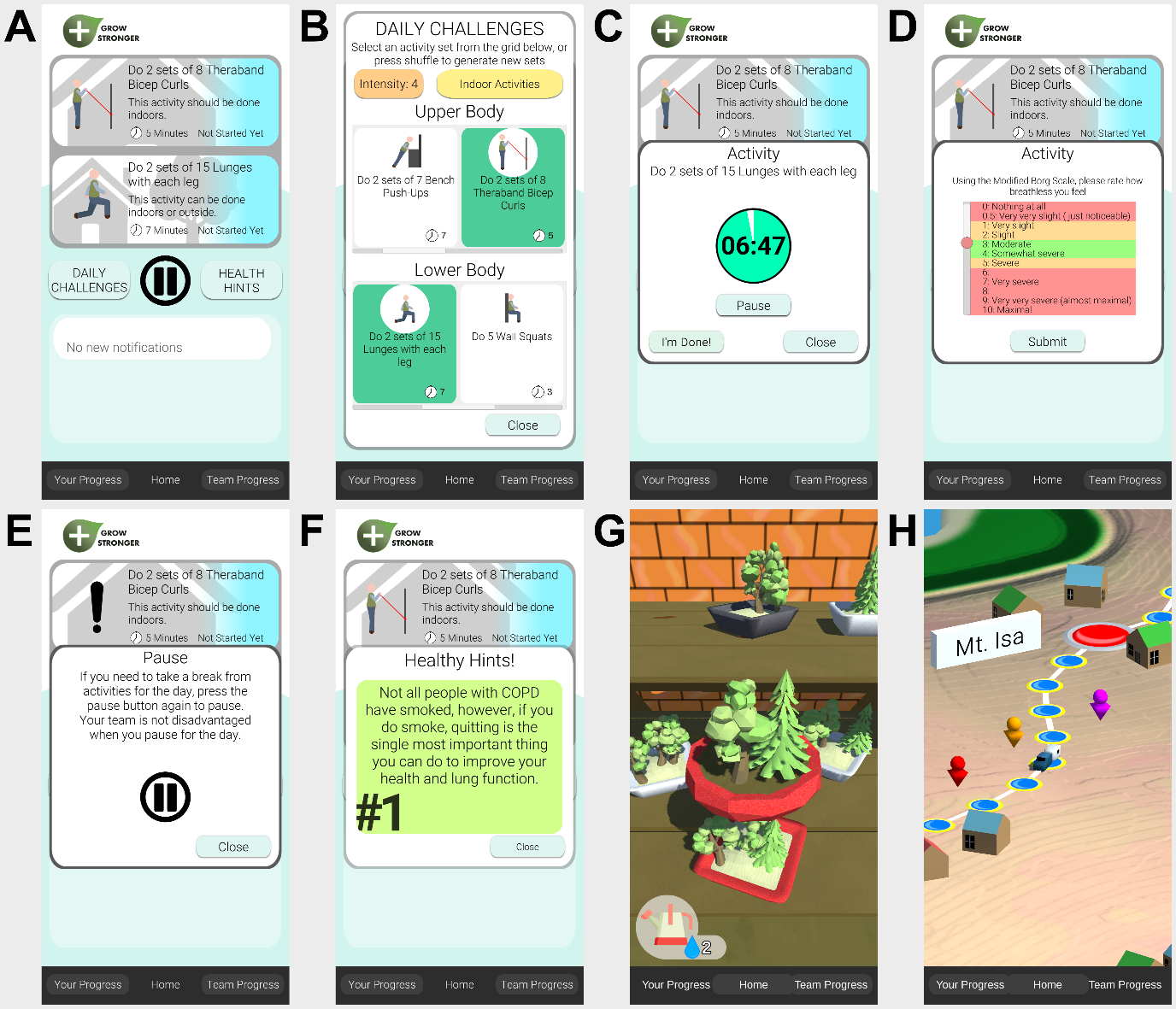


(A) Home screen. From this screen, other menus can be selected, notifications can also be seen on this screen, and the game can be paused if unable to participate; (B) Daily challenges menu. Two activities were chosen each day. These can be filtered indoors or outdoors (or both), and the intensity can be selected; (C) Exercise timer, which starts when the exercise begins and can be paused and resumed or stopped. These times are recorded in the database; (D) Breathlessness rating. Immediately after completing an activity, players are asked to move the slider to best represent how breathless they feel; (E) Pause menu. On hitting the pause button, players are given this prompt explaining the purpose of the pause and confirming their wish to pause. The pause can be resumed by hitting the pause button once more; (F) Healthy hints. A new piece of information relevant to COPD is presented each day; (G) Individual progress. Themed as a garden, where drops of water can be earned by completing daily challenges and can be used to grow plants. (H) Team progress. A team cooperates on a caravan trip around Australia. The trip progresses based on the average number of daily challenges completed by the team each day. Colored pieces representing other team members may be displayed next to the caravan.

As seen in the screenshots, the game features two parallel and independent game modes. The first mode functions as a single player mode and uses the theme of growing a garden. In this mode, the completion of physical activity tasks will reward the player with water in a watering can that can be used to grow a potted plant. The second game mode functions as a co-operative multiplayer game mode and has the theme of a caravan trip around Australia, visiting multiple well-known Australian destinations. In this mode, the team’s progress, represented by a caravan, is determined by the average number of activities completed by the team. As a form of social interaction during the caravan trip, a random sample of 3 to 4 activities completed by other players in the last 24 hours will be displayed next to the caravan as represented by individual player pieces. By selecting these pieces, a player can send a pre-set *“cheer”* message to the other player whose activity is represented by that piece to congratulate them on the achievement of that activity.

Players are restricted to performing one upper-body and one-lower body activity per day, as clinicians involved in the co-design process were concerned that providing rewards for activity could encourage some participants to exert themselves too much by doing as much as possible in a given day, rather than adequately pace themselves. Therefore, the game was focused on building a habit of regularly performing activities rather than performing as many activities as possible.

The game is designed to work in concert with clinicians to provide a level of supervision over the players. All data from the use of the game is reported to a web interface which allows the clinicians to monitor the progress of all players. In addition, the clinician interface allows for exercises to be restricted to only some player. This feature was requested by clinicians during the co-design process, in order to avoid exercises that might be contra-indicated from appearing in the list of available options. Clinicians can also use this interface to send messages to any individual player, including pre-set messages of encouragement.

The game also provides health information in the form of short snippets known in the game as ‘*healthy hints’*. These ‘*healthy hints*’ are presented once daily and consist of a variety of short informational messages relevant to living with COPD, derived from publicly available information booklets published by health organisations in Australia.

The following table details all the available activities in *Grow Stronger*:

| ***Category*** | ***Intensity 1*** | ***Intensity 2*** | ***Intensity 3*** | ***Intensity 4*** |
| --- | --- | --- | --- | --- |
| *Sit-to-Stands* | 5 reps | 10 reps | 2 sets of 10 reps | 2 sets of 12 reps |
| *Walk (Indoors)* | 2 minutes | 5 minutes | 10 minutes | 15 minutes |
| *Walk (Outdoors)* | 2 minutes | 5 minutes | 10 minutes | 15 minutes |
| *Wall Pushups* | 2 sets of 2 reps | 2 sets of 4 reps | 3 sets of 5 reps | 4 sets of 6 reps |
| *Lunges* | 5 reps | 10 reps | 2 sets of 10 reps | 2 sets of 15 reps |
| *Bench Push-Ups* | 3 reps | 6 reps | 2 sets of 5 reps | 2 sets of 7 reps |
| *Wall Squats* | 2 reps | 3 reps | 4 reps | 5 reps |
| *Step-Ups* | 5 reps | 8 reps | 2 sets of 6 reps | 2 sets of 9 reps |
| *Theraband Bicep Curls* | 4 reps | 7 reps | 2 sets of 5 reps | 2 sets of 8 reps |
| *Theraband Arm Extensions*  *(Shoulder Extension)* | 4 reps | 6 reps | 2 sets of 5 reps | 2 sets of 8 reps |
| *Theraband Arm Forwards (Shoulder Flexions)* | 4 reps | 6 reps | 2 sets of 5 reps | 2 sets of 8 reps |
| *Theraband Standing Row* | 2 reps | 3 reps | 4 reps | 5 reps |
| *Triceps Bench Dips* | 2 reps | 4 reps | 7 reps | 2 sets of 5 reps |
| *Rear-Foot Elevated Lunge (Split Squats)* | 2 reps | 4 reps | 6 reps | 2 sets of 5 reps |
| *Bouncing a Ball* | 2 minutes | 5 minutes | 10 minutes | 15 minutes |

Reps, repetitions.
