# Supplementary material for: A Co-Designed Active Video Game for Physical Activity Promotion in People With Chronic Obstructive Pulmonary Disease: Pilot Trial
Source: JMIR Serious Games. 2021 Jan 27;9(1):e23069. doi: 10.2196/23069 (PMC7875701; doi:10.2196/23069)
Supplement: Multimedia Appendix 2 [file games_v9i1e23069_app2.docx]

# Multimedia Appendix 2

(A) Daily step counts for the experiment group.


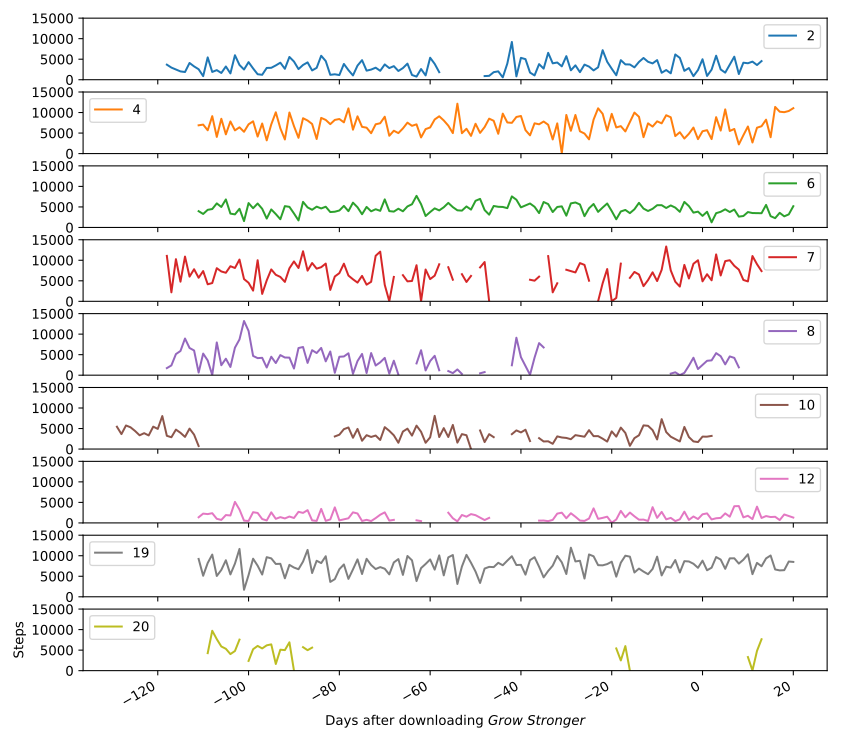
 Blank sections indicate a lack of Fitbit data for that time point.

(B) Daily step counts for the control group.


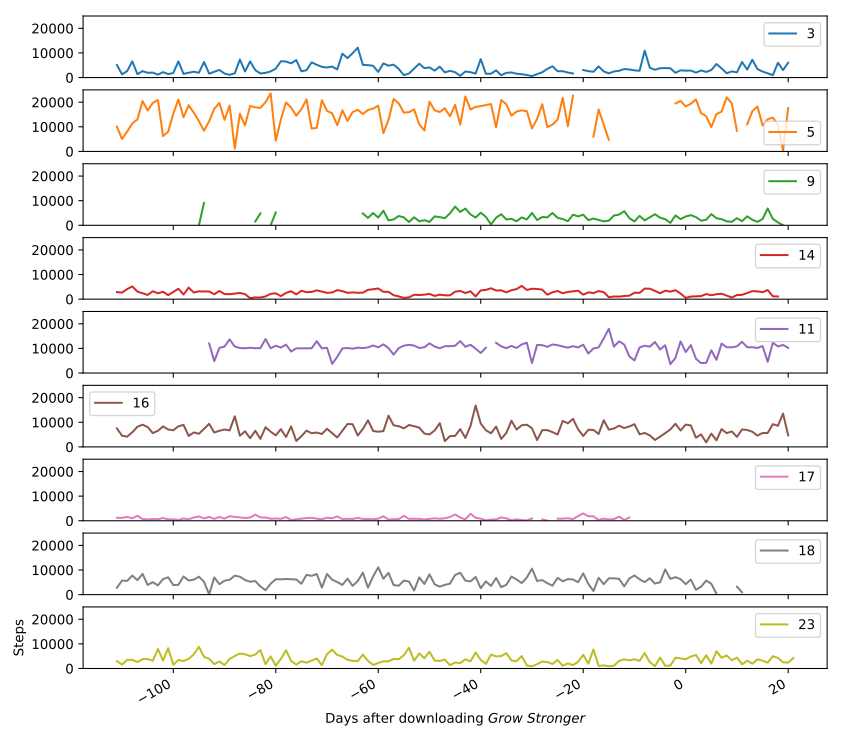


Blank sections indicate a lack of Fitbit data for that time point.
