# Supplementary material for: A Co-Designed Active Video Game for Physical Activity Promotion in People With Chronic Obstructive Pulmonary Disease: Pilot Trial
Source: JMIR Serious Games. 2021 Jan 27;9(1):e23069. doi: 10.2196/23069 (PMC7875701; doi:10.2196/23069)

# Multimedia Appendix 3

A scatter matrix for correlations between the primary and secondary outcome measures in the experiment group.


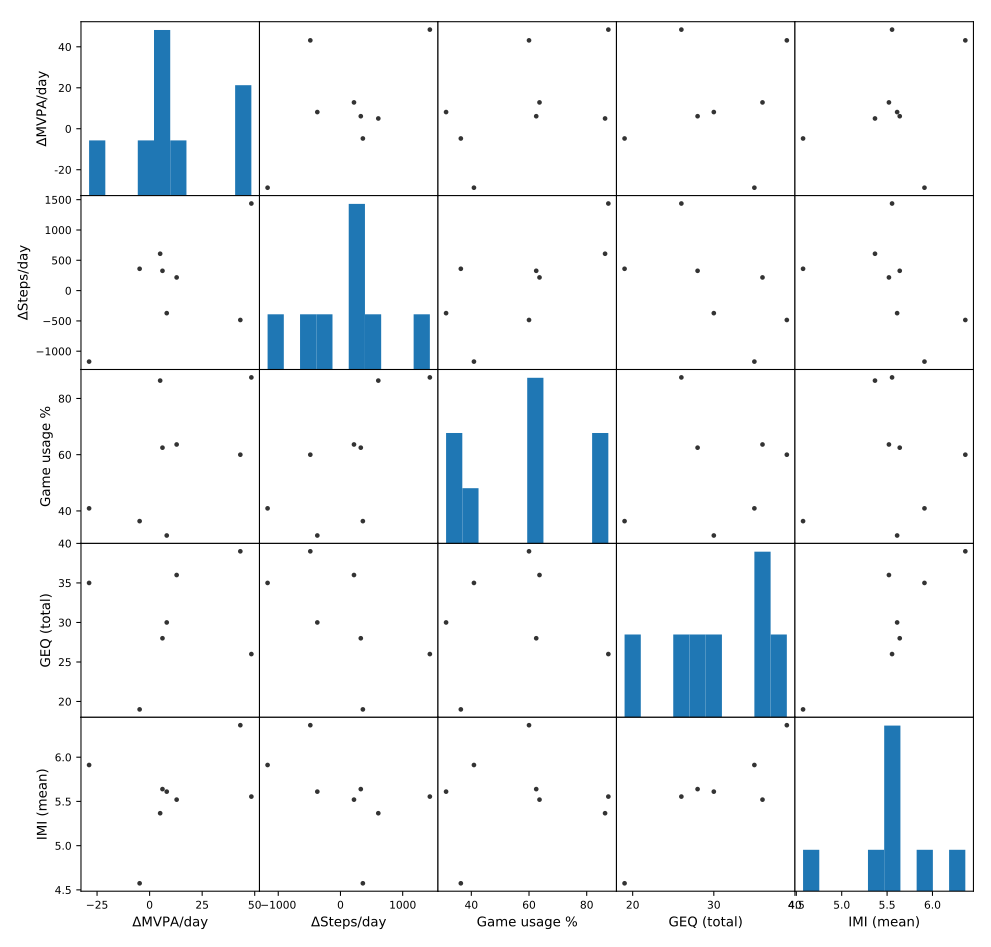

Supplement: Multimedia Appendix 3 [file games_v9i1e23069_app3.docx]
